# Global gene expression profiling for fruit organs and pathogen infections in the pepper, *Capsicum annuum* L.

**DOI:** 10.1038/sdata.2018.103

**Published:** 2018-06-05

**Authors:** Myung-Shin Kim, Seungill Kim, Jongbum Jeon, Ki-Tae Kim, Hyun-Ah Lee, Hye-Young Lee, Jieun Park, Eunyoung Seo, Saet-Byul Kim, Seon-In Yeom, Yong-Hwan Lee, Doil Choi

**Affiliations:** 1 Interdisciplinary Program in Agricultural Genomics, Seoul National University, Seoul 08826, Korea; 2 Department of Plant Science, Plant Genomics and Breeding Institute, Research Institute for Agriculture and Life Sciences, Seoul National University, Seoul 08826, Korea; 3 Department of Agricultural Biotechnology, Seoul National University, Seoul 08826, Korea; 4 Division of Eco-Friendly Horticulture, Yonam College, Cheonan 31005, Korea; 5 Department of Agricultural Plant Science, Division of Applied Life Science (BK21 Plus), Institute of Agriculture & Life Science, Gyeongsang National University, Jinju 52828, Korea

**Keywords:** Transcriptomics, Plant immunity, Plant domestication, RNA sequencing, Gene expression

## Abstract

Hot pepper (*Capsicum annuum*) is one of the most consumed vegetable crops in the world and useful to human as it has many nutritional and medicinal values. Genomic resources of pepper are publically available since the pepper genomes have been completed and massive data such as transcriptomes have been deposited. Nevertheless, global transcriptome profiling is needed to identify molecular mechanisms related to agronomic traits in pepper, but limited analyses are published. Here, we report the comprehensive analysis of pepper transcriptomes during fruit ripening and pathogen infection. For the ripening, transcriptome data were obtained from placenta and pericarp at seven developmental stages. To reveal global transcriptomic landscapes during infection, leaves at six time points post-infection by one of three pathogens (*Phytophthora infestans*, *Pepper mottle virus*, and *Tobacco mosaic virus* P0 strain) were profiled. The massive parallel transcriptome profiling in this study will serve as a valuable resource for detection of molecular networks of fruit development and disease resistance in *Capsicum annuum*.

## Background and Summary

Large amounts of transcriptome data have been released using next-generation sequencing technology for past decades, which enables us to study organisms in a genomic perspective. In plants, global gene expression profiling was performed to elucidate molecular mechanisms for organ specificity, developmental changes, and disease resistance^1–10^. For example, the transcriptome analysis on developing seeds suggested that transcriptional change in endosperm and embryo was regulated by distinct co-expressed networks in wheat and maize^1,2^. In addition, the expression analysis of pathogen infected leaves in Arabidopsis and tomato revealed that a number of genes and networks interacted with each other in a specific time and a stage^7–10^. A recent study using multiple transcriptomes identified the vacuolar protease SLVPE3 and their target, serine protease inhibitor KTI4, involved in fruit ripening and disease resistance^11^. These genomic and transcriptomic studies have allowed us to unveil gene expression mechanisms and find target genes associated with agronomic traits.

Hot peppers (*Capsicum* spp.), belonging to Solanaceae family, are the most widely cultivated spice in the world. In 2013, the worldwide production of pepper was 31.1 million tons (14.6 billion US dollars), which was the third largest among vegetable crops^12^. The pepper fruits are rich sources of vitamin C, pigments, minerals and pungent agents that are known as nutritional and functional properties for human health^13^. The genus *Capsicum* consists of 33 undomesticated and five domesticated species including the most widely cultivated species, *Capsicum annuum*^14^. Various genetic studies for the pepper have been performed to unveil molecular mechanisms of important agronomic traits and disease resistance^15–24^. Recently, completion of the multiple reference pepper genomes with the deposited large amount of transcriptome data has enabled to perform in-depth analyses for these agronomical traits^13,25–28^. However, comprehensive transcriptome analyses to identify expression and expressional variations of genes using the large transcriptome resources of the peppers are still lacking.

**Figure 1:**
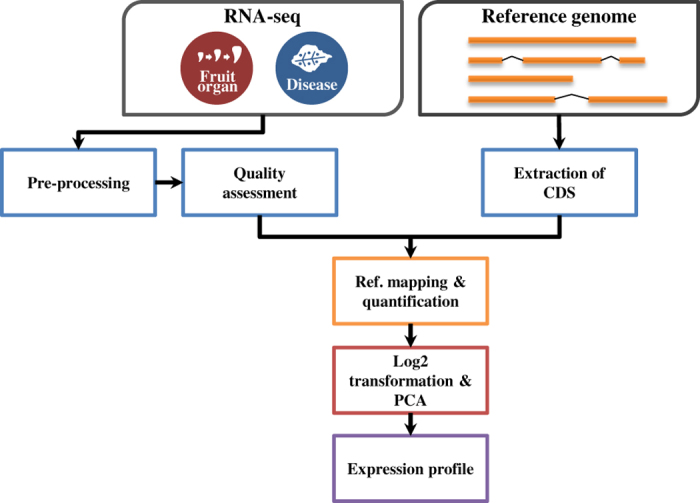
Schematic overview of the analysis pipeline. The pepper transcriptome of fruit organs and pathogen- infected leaves including three biological replicates except for Mock-Up (n=2) were collected from NCBI SRA (SRP106410 and SRP119199). All raw sequences were pre-processed and assessed using FastQC (http://www.bioinformatics.babraham.ac.uk/projects/fastqc) and MultiQC. The filtered reads were mapped to *Capsicum annuum* reference genome (v.1.55) using CLC assembly. The mapped reads were normalized RPKM and log2 transformed mean value were used to PCA.

**Figure 2:**
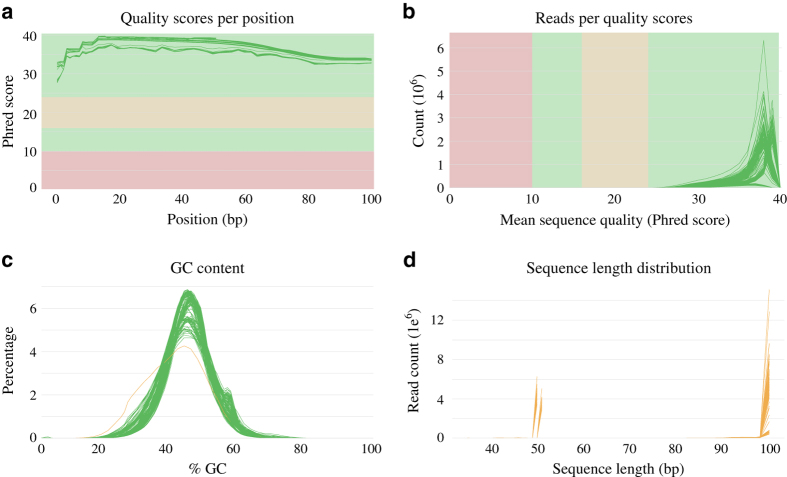
Quality assessment of pepper transcriptomes. The filtered reads from all 136 samples were assessed by MultiQC. (**a**) Mean quality scores distribution in each position. (**b**) Read counts distribution for mean sequence quality. (**c**) GC ratio distribution. (**d**) Read length distribution.

**Figure 3:**
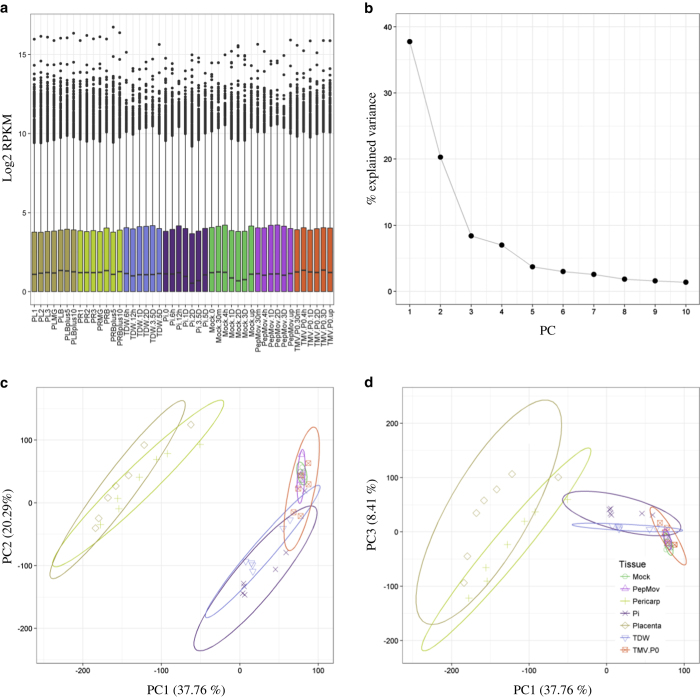
Global gene expression pattern in pepper transcriptomes. The log2 transformed mean RPKM values were plotted by boxplot function in R (**a**). The line plot (**b**) and scatter plots of PC1 versus PC2 (**c**) and PC1 versus PC3 (**d**) were drawn using previously published code with modification^32^. The abbreviations see method section 2.

In this study, we openly released the hot pepper transcriptomes that were previously published^13,21,23^. We described in detail the expression profiling methods of samples from fruit development, pathogen infection in each time point and tissues in *C. annuum* (Fig. 1). Total 125.68 Gb of transcriptome data from previously reported fruit tissues (pericarp and placenta) and infected leaves with *P. infestans, Pepper mottle virus (PepMov)*, and *Tobacco mosaic virus* (*TMV)* P0 strain was generated (Table 1 and Data Citation 1). After preprocessing analyses, we mapped the remaining sequences to the reference pepper genome (Data Citation 1). The preprocessed sequences were validated through quality assessment (Fig. 2). A principal component analysis (PCA) showed the global gene expression patterns and variations between samples (Fig. 3). Consequently, the expression profiling of multiple conditions in pepper will provide valuable resources for analysis on fruit development, ripening and disease resistance.

## Methods

### Experimental overview

Massive transcriptome data for seven developmental stages in fruit (fruit development set) and six to seven time points in leaves infected by pathogens (pathogen infection set) were generated to decipher global gene expression profiling for fruit development and disease resistance in *C. annuum*. The reference pepper genome annotation v1.55 was used (http://peppergenome.snu.ac.kr). Reference mapping and normalization for filtered transcriptome were performed after quality filtering and assessment. A principal component analysis (PCA) was performed to elucidate global gene expression patterns and evaluate the correlation between samples using log2 transformed RPKM values (Fig. 1).

### Transcriptome data generation

The transcriptome data in this study were acquired from CM334 dataset (Data Citation 2 and Data Citation 3). For transcriptome profiling of fruit development, pepper fruits at seven ripening stages were harvested at 6, 16, 25, 36, 38, 43, and 48 days post-anthesis (DPA) as previously described^13^. For transcriptome profiling of immune response to multiple pathogens, pepper leaves were inoculated with 15 μl droplets of 5×10^4^ zoospores ml^−1^ suspension in *P. infestans*, and *PepMov* and *TMV* P0 strain purified from systemically infected tobacco leaves as previously described^21,23^. Inoculated leaves harvested at several time points from three biological replicates were ground in liquid nitrogen, which was used for total RNA purification. The strand-specific libraries with 150–200 bp insert size were constructed and sequenced with Illumina HiSeq 2000 and 2500 platforms (Illumina Inc., San Diego, USA) using fruit development set and pathogen infection set, respectively. Sample names were assigned: placenta (PL); pericarp (PR); stage 1, 6 DPA (1); stage 2, 16 DPA (2); stage 3, 25 DPA (3); mature green, 36 DPA (MG); breaker, 38 DPA (B); breaker plus 5, 43 DPA (B5); and breaker plus 10, 48 DPA (B10); control for *P. infestans* (TDW) and virus (Mock); infection for *P. infestans* (Pi), pepper mottle virus (PepMov), TMV P0 strain (TMV). Only single (forward) reads were used in pathogen infection set to reduce the read type variable for the fruit development set.

### Pre-processing and quantification

The raw sequences of transcriptome were filtered and trimmed using previously described methods to remove contaminated and low quality reads^13^. The raw reads containing reference bacterial sequences were filtered using Bowtie2 v2.0.0-beta7 with modified parameters (--local –D 15 –R 2 –N 0 –L 20 –I S,1,0.65)^29^. The sequences with quality scores below 20 were trimmed using the CLC quality trimming software (CLC bio, Aarhus, Denmark). Minimum length cut-off for 50 bp and 101 bp read was 35 bp and 71 bp, respectively. The reads were validated using FastQC v0.11.5 (ref. 30) and MultiQC v1.3.dev0 (ref. 31) software with default parameters. The processed reads were mapped to the v.1.55 pepper CDS using CLC assembly cell with –s 0.99 –l 0.9 parameters (CLC bio, Aarhus, Denmark). Total mapped reads were normalized to reads per kilobase per million mapped reads (RPKM).

### Principal component analysis (PCA)

Average RPKM values for each time point and tissue were used for PCA. To reduce the influence of extremely expressed genes, RPKM values were log2-transformed and boxplot was drawn using boxplot function in R. PCA was performed using previously published code with modification^32^.

## Data Records

The detailed transcriptome information and average RPKM values for all pepper samples were deposited in figshare (Data Citation 1). The raw reads for transcriptome were deposited in NCBI Sequence Read Archive (SRA) accession (Data Citation 2 and Data Citation 3).

## Technical Validation

### Quality validation

To assess total data quality, we performed the quality check using FastQC and MultiQC software for all preprocessed samples. Overall, the mean quality scores in each base position were higher than 27 (Fig. 2a). The read counts per quality scores were distributed above 25 and average quality was higher than 35 (Fig. 2b). The normal distribution of GC content was indicating non-contaminated in sequencing process (Fig. 2c). The average sequence lengths were 50 bp and 99 bp for fruit development set and pathogen infection set, respectively (Fig. 2d). These numerical values represent that high-quality sequences were obtained for further analysis.

### Global gene expression analysis

To elucidate global gene expression patterns in multiple conditions, filtered reads were mapped to pepper CDS and normalized by RPKM. The average RPKM values of three biological replicates in each sample were used for further analysis. A principal component analysis using log2 transformed RPKM showed that first three PCs explained most of the variance (Fig. 3a,b). The comparisons between PC1 and PC2 or PC3 indicated that the group of fruit organs and leaves infected by pathogen were separated clearly. In addition, the leaves infected by *P. infestans* and group of virus (PepMov and TMV P0 strain) showed a different pattern with minor overlap. (Fig. 3c,d).

## Additional information

**How to cite this article:** Kim M.-S. *et al.* Global gene expression profiling for fruit organs and pathogen infections in the pepper, *Capsicum annuum* L. 5:180103 doi: 10.1038/sdata.2018.103 (2018).

**Publisher’s note:** Springer Nature remains neutral with regard to jurisdictional claims in published maps and institutional affiliations.

## Supplementary Material



## Figures and Tables

**Table 1:** Statistics of pepper transcriptomes used in this study.

Sample	Tissue/treatment	Read type	Sampling method	Time point	Preprocessed data (Gb)	Accession number
Fruit organ	PlacentaPericarp	Single	Tissue sampling	6, 16, 25, 36, 38, 43, 48 DAP	4.325.12	SRP119199
Oomycete	*P. infestans*TDW	Paired	Suspensiondroplet	0, 6, 12, 2448, 90, 120 h	13.210.92	SRP106410 SRP119199
Virus	PepMovTMV_P0Mock	Paired	Rubbing with carborundum on the leaves	0, 0.5, 4, 24, 48, 72 h and systemic leaves	9.666.1515.16	SRP119199
PepMov: pepper mottle virus; TMV_P0: tobacco mottle virus P0 strain; TDW: control for *P. infestans*; Mock: control for viruses; DPA: days post-anthesis.						
